# Multivariate Adaptability of Tropical Wheat Cultivars to Drought and Salinity Stresses

**DOI:** 10.3390/plants14071021

**Published:** 2025-03-25

**Authors:** Alan Mario Zuffo, Jorge González Aguilera, Francisco Charles dos Santos Silva, Ricardo Mezzomo, Leandra Matos Barrozo, Fábio Steiner, Bruno Rodrigues de Oliveira, César Augusto Masgo Soto, Carlos Genaro Morales-Aranibar, Nataniel Linares-Gutiérrez, Luis Morales-Aranibar

**Affiliations:** 1 Department of Agronomy, State University of Maranhão, Balsas 65800-000, MA, Brazil; franciscocharlessilva@professor.uema.br (F.C.d.S.S.); ricardomezzomo@professor.uema.br (R.M.); leandrabarrozo1@gmail.com (L.M.B.); 2 Department of Crop Science, State University of Mato Grosso do Sul, Cassilândia 79540-000, MS, Brazil; jorge.aguilera@uems.br (J.G.A.); steiner@uems.br (F.S.); 3 Editora Pantanal, Nova Xavantina 78690-000, MT, Brazil; bruno@editorapantanal.com.br; 4 Department of Environmental Engineering, National University of Engineering (UNI), Lima 15333, Peru; cmasgo@uni.edu.pe; 5 Faculty of Sciences, National University of Engineering, Tupac Amaru Av. 210, Rimac, Lima 15333, Peru; cgmoralesa@unjbg.edu.pe; 6 Faculty of Engineering, Jorge Basadre Grohmann National University, Tacna 23001, Peru; nlinaresg@unjbg.edu.pe; 7 Department of Civil Engineering and Basic Sciences, National Intercultural University of Quillabamba, Cusco 08741, Peru; luis.morales@uniq.edu.pe

**Keywords:** abiotic stress, osmotic stress, water stress, salt stress, *Triticum aestivum*

## Abstract

Wheat production in the Brazilian Cerrado region faces challenges related to drought and salinity, which limit plant development and crop yield. This study evaluated the multivariate adaptability of 11 tropical wheat cultivars to drought and salinity stresses during early plant development. Wheat plants were grown for 12 days at 25 °C under non-stressful (control) and simulated drought and salinity stress conditions with –0.30 MPa iso-osmotic solutions prepared with polyethylene glycol or sodium chloride, respectively. The germination, growth rate and dry matter accumulation of the plants were measured. The results showed that wheat cultivars have distinct morphological responses to stressful environmental conditions, with drought stress having a greater impact on shoot growth and saline stress having a greater impact on root system development. The multivariate adaptability and stability analyses performed using the Lin and Binns method and GGE biplot revealed that the wheat cultivars BIO 190057, BRS 404 and TBIO Duque combine adaptability and stability for all morphological traits simultaneously, being considered cultivars tolerant to drought and salinity stresses. It was concluded that the identification of cultivars tolerant and adapted to adverse environmental conditions is essential for the advancement of sustainable cultivation of tropical wheat in the Brazilian Cerrado region, contributing to global food security.

## 1. Introduction

Wheat (*Triticum aestivum* L.) production in Brazil’s Cerrado region presents challenges and opportunities. Drought and salinity tolerance are crucial to unlocking the production potential in this agricultural region, where limited water availability during the growing season impairs yield [1] and is a challenge for wheat production.

The Cerrado has emerged as a new agricultural frontier for tropical wheat cultivation, where some genotypes have shown promising technological qualities suitable for bakery products [2], among other traits. The constant search for new genotypes with high yield potential and adapted to adverse environmental conditions is the focus of numerous research projects in Brazil and worldwide.

Wheat in the Cerrado faces significant challenges due to water restriction stress, which inhibits plant growth and production [3]. To address this issue, researchers are focusing on developing drought-tolerant wheat varieties through several approaches. Low soil water availability also affects wheat production in other tropical regions of the world, where water resources are scarce or insufficient [4,5]. Severe and prolonged drought periods cause significant decreases in crop yield and recent climate change has contributed to the more frequent emergence of abiotic stresses, which can compromise food security [4,6].

Morphological and physiological traits have been widely used to identify drought-tolerant genotypes [3,7]. Biochemical, molecular and genetic traits have also been explored to improve the adaptation of wheat genotypes to adverse environmental conditions [8,9]. Studies on the identification of tolerant genes and the combined capacity of plant morphophysiological traits have been used to improve wheat grain yield under drought stress conditions [7]. The genetic characterization of Brazilian wheat breeding materials is important to reveal the differences between the tropical cultivars grown in the Cerrado region and the winter cultivars recommended for the southern region of Brazil [8,9]. These multifaceted approaches aim to develop drought-tolerant wheat genotypes to improve the sustainability of national wheat production.

In addition to drought stress, wheat faces significant challenges due to salinity stress caused by excess soluble salts in the soil solution, which negatively impacts plant development and crop yield [10,11,12]. Salinity affects several physiological and biochemical processes in wheat plants, including enzymatic activity, photosynthesis and numerous other metabolic reactions [13]. Some research has been conducted to improve understanding of salinity stress tolerance mechanisms of wheat plants. These studies include conventional breeding, molecular techniques, and the utilization of genetic diversity from wild relatives [11,12]. Sustainable wheat production in saline fields can be achieved through a combination of biotechnological, biological and biochemical methods along with proper irrigation management [13,14].

In the current scenario of Brazilian and global agricultural production with recent climate change, extreme drought events and soil salinization, research aimed at identifying wheat genotypes that are tolerant and adapted to abiotic stress conditions is essential to improve wheat production and food supply. In these areas of research, univariate or multivariate selection methods need to assist in the process of identifying superior genotypes in adverse environmental conditions.

This study aimed to evaluate the multivariate adaptability of 11 tropical wheat cultivars to drought and salinity stresses during early plant development.

## 2. Material and Methods

### 2.1. Plant Material and Stress Treatments

This study was conducted at the Seed Laboratory, State University of Maranhão (UEMA), in Balsa, MA, Brazil. Seeds from 11 tropical wheat cultivars (*Triticum aestivum* L.) commonly grown in the Brazilian Cerrado region were used in this study. Before starting the experiment, the water content, 1000-seed weight, seed germination rate and plant emergence rate were determined following the Official Rules for Seed Analysis [15]. The main characteristics of wheat seeds and cultivars are shown in Table 1.

To compare the effects of drought and salinity stress on germination rates and plant growth rates, the seeds of each wheat cultivar were exposed to 0.30 MPa iso-osmotic solutions with polyethylene glycol (PEG-6000) and sodium chloride (NaCl), respectively. The amount of PEG-6000 added to obtain the solution with an osmotic pressure of −0.20 MPa was determined by the equation of Michel & Kaufmann [16]: Ψ_S_ = [−(1.18 × 10^−2^) C—(1.18 × 10^−4^) C^2^ + (2.67 ×10^−4^) CT + (8.39 × 10^−7^) C^2^T]/10, where Ψs is the osmotic potential (MPa); C is the concentration (g L^−1^ PEG-6000); and T is the temperature (°C). The amount of NaCl added to obtain an osmotic pressure of −0.30 MPa was calculated by the van’t Hoff equation [17]: Ψ_S_ = −*R* × *T* × *C* × *i*, where *R* is the ideal gas constant (0.008314 MPa mol^−1^ K^−1^); *T* is the absolute temperature (273.15 + °C); *C* is the concentration in molarity of the solute (mol L^−1^); and *i* is the van’t Hoff factor, that is the number of ions released when the solute is dissolved in water [i.e., for NaCl this value is 2.0 (Na^+^ and Cl^−^). Distilled water with an osmotic potential of 0.00 MPa was used as the control treatment.

### 2.2. Germination and Growth Conditions

Four 25-seed replicates from each wheat cultivar were evenly distributed between two sheets of paper towels, previously moistened with distilled water (control), PEG-6000 solution (drought stress) or NaCl solution (salinity), in an amount equivalent to three times the dry mass of the Germitest^®^ paper. The paper towel sheets were then turned into rolls, which were packaged into plastic bags to prevent evaporation and maintain the relative humidity close to 100%. Germination was conducted in a growth chamber under a 12/12 h photoperiod (light/darkness), light intensity of 120 μmol m^−2^ s^−1^ and a temperature of 25 °C for 12 days. Vitavax–Thiram^®^ (carboxine + thiram) was added to the solutions at a concentration of 0.2% (*v*/*v*) to control the fungal infection as used by Zuffo et al. [18]. Plants remained exposed to abiotic stress for 12 days. In a previous trial, a period of 12 days was appropriate to identify differences in tolerance between wheat genotypes exposed to osmotic stress conditions, especially since this period is ideal for radicle and epicotyl elongation, two processes that are delayed when the germination environment has low osmotic potential.

### 2.3. Measurement of Germination, Plant Growth and Tolerance Indices

After 12 days of exposure to abiotic stress, the seed germination rate was recorded. Seeds were considered germinated when the radicle was longer than 10.0 mm. Subsequently, five plants per replicate were randomly chosen and the shoot length (SL), root length (RL), total plant length (TL), shoot dry matter (SDM), root dry matter (RDM) and total dry mass (TDM) were determined. The SL, RL and TL were measured using a millimeter ruler. The SDM, RDM and TDM were recorded on an analytical balance after oven drying at 85 °C for 48 h.

### 2.4. Experimental Design and Statistical Analyses

The experiment was arranged in a completely randomized design, with a 3 × 11 factorial design, with three osmotic stress treatments (control, drought and salinity stress) and 11 tropical wheat cultivars, with four replications.

First, individual analysis of variance for each environmental condition followed by joint analysis of variance was performed according to the statistical model described in Equation (1):

Y_ijk_ = μ + G_i_ + E_j_ + G_i_ × E_ij_ + e_ijk_ (1)

where Y_ijk_ is the observation assessed in the i-th genotype and j-th environment; µ is the overall mean of the tests; G_i_ is the effect of the i-th genotype considered fixed; E_j_ is the effect of the j-th environment considered random; G_i_ × E_ij_ is the random effect of the interaction between genotype i and environment j; and e_ijk_ is the random error associated with the observation Y_ijk_. We demonstrated the difference between each abiotic stress using a box plot for each dependent variable measured in wheat plants [i.e., G, SL, RL, TL, SDM, RDM and TDM].

For each plant morphological trait, multivariate adaptability and stability were evaluated using the Lin and Binns method [19]. In this method, the overall recommendation is made based on the lowest *Pi* estimates for each trait, according to Equation (2):

(2) $$P_{i}=\frac{\sum_{j=1}^{e}(Y_{ij}-Y_{mj})2}{2e}$$

where *Y* is the value of the trait of the *i*-th genotype in the *j*-th environment and *Y* is the estimation of the trait of the best genotype in environment *j.*

In turn, the multivariate parameters of adaptability and stability, while considering all the traits for the set of environments (*P*), were estimated according to Equation (3), proposed by Aguilera et al. [20]:

(3) $$P_{im}=\sum_{k=1}^{v}\left[\frac{P_{ik}}{{\hat{\sigma }}_{Pik}}\right]$$

where *P* is the univariate estimator of the adaptability and stability of the i-th genotype associated with the *k*-th variable; *P* is the univariate estimator of the adaptability and stability of the *i*-th genotype in favorable environments associated with the *k*-th variable; and *P* is the standard deviation of *P*. Finally, a heatmap was built to graphically show the results of univariate and multivariate analyses.

Finally, to ensure greater robustness in the conclusions, the adaptability and stability of the genotypes were also estimated using the GGE biplot method through a multivariate approach. To analyze all the variables simultaneously, the scores of the first principal component obtained from the principal component analysis were used as variables in the GGE analysis.

The adopted GGE biplot model is defined by Equation (4):

(4) $$Y_{ij}-y_{j}=y_{1}\varepsilon_{i}\rho_{j1}+y_{2}\varepsilon_{i}\rho_{j2}+\varepsilon_{ij}$$

where *Yij* represents the average grain yield of genotype *i* in the environment. Yi is the general mean of the genotypes in environment *j*; y1 εi1ρj1 is the first principal component (PC1); y2 εi2ρj2 is the second principal component (PC2); y1 and y2 are the eigenvalues associated with the principal component analysis PCA1 and PCA2, respectively; ε1 and are the PC1 and PC2 scores, respectively, for genotype *i*; ρj1 and ρj2 are the PC1 and PC2 scores, respectively, for environment *j*; and *εij* is the error associated with the model for the *i*-th genotype and *j*-th environment [21]. All analyses were performed using the Rbio^®^ software version 140 for Windows [22].

## 3. Results

There was a highly significant effect (*p* < 0.001) of genotype (G) and environment (E) on all morphological traits of wheat plants (Table 2). The GxE interaction was significant only for the RL (*p* < 0.001) and SDM (*p* < 0.05), indicating that the differential expression of genotypes occurred in response to abiotic stress conditions. The coefficients of variation obtained in the joint analysis were less than 12% for all morphological traits. These findings indicate high experimental precision, showing high homogeneity in the data obtained for the different experimental conditions evaluated.

**Figure 1:**
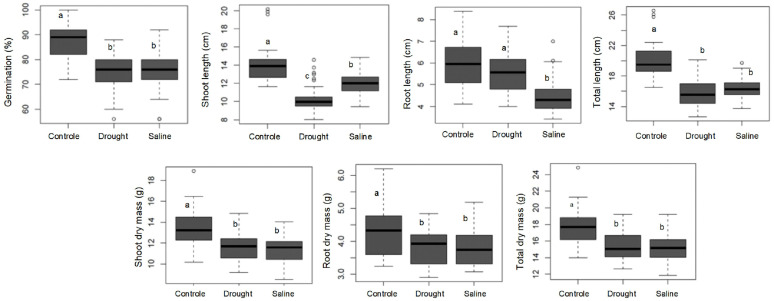
Effects of non-stressful (control) and stressful (drought and salinity) growth environments on the germination, shoot length, root length, total plant length, shoot dry mass, root dry mass and total dry mass of 11 tropical wheat cultivars (*Triticum aestivum* L). The total number of samples (n) for each environment was 44. Bars followed by different letters show significant differences using the Tukey test (α = 0.05).

The non-stressful condition (control) resulted in the highest values for all plant morphological traits, confirming that abiotic stresses (drought and salinity) limited germination and initial growth of wheat cultivars (Figure 1). The impact of drought and salinity stresses on seed germination was similar, with a 15% reduction in wheat germination rate compared to the control treatment, reaching average values of 75%.

A similar response was observed for shoot dry matter and total dry matter (Figure 1), showing that the impact of drought and salinity stresses are similar for plant dry matter accumulation. Drought stress did not affect root length; however, this stress drastically reduced shoot growth of wheat plants.

In general, drought stress had a more severe impact on shoot length, highlighting the detrimental effects of drought on shoot growth. In turn, saline stress had a more severe impact on root length and root dry matter, limiting the growth of the plant root system due to osmotic and ionic stress caused by the high concentration of soluble salts in the soil solution (Figure 1).

The application of the Lin and Binns method allowed us to evaluate the adaptation capacity and stability of the tropical wheat cultivars (Table 3). The method defines the most adapted genotype as the one with a lower value when calculating the univariate index for each measured variable for each of the abiotic stress environments (D: drought and S: salinity), compared to the non-stressful environment (control). For seed germination, the three best cultivars were TBIO Aton, TBIO Calibre and TBIO Duque, with values *Pi* of 0, 0.71 and 0.9, respectively. TBIO Aton and TBIO Calibre stand out in both stresses as being those that show the least variation in relation to the control treatment (Table 3). The genotype BIO 190057 shows a large variation in response to drought (58) and salinity stress (128), evidencing a differential effect of the two stresses applied in relation to the control treatment.

Regarding the shoot length, the cultivars BIO 190057, BRS 264 and BRS 404 were superior with values of 0.03, 0.04 and 0.10, respectively. In this dependent variable, no cultivar of those evaluated was found with large variations in relation to the stress, which indicates that the variable does not undergo many variations when both stresses are applied.

In the case of the root length, the genotypes ORS Feroz, TBIO Sossego and TBIO Calibre showed greater root growth, with values of 0.06, 0.13 and 0.29, respectively. For total plant length, the cultivars BIO 190057, BRS 264 and BRS 404 were superior with values of 0.04, 0.02 and 0.15, respectively. For the shoot, root and total dry mass, the cultivars BIO 190038 and BIO 190057 were superior. Total length and dry matter accumulation of shoots, roots and total are variables that show little variation among wheat cultivars when considering both abiotic stresses (Table 3). The only highlight is the TBIO Convicto cultivar, which in total length had a decrease by half from water stress to salinity stress, with values of 6 to 3, respectively. On the other hand, the root dry mass showed the lowest values among all the variables, which shows a greater effect of the variations between the applied stresses.

In the *Pim* multivariate analysis, the genotypes BIO 190057, BRS 404 and ORS Feroz obtained the lowest indexes (3.08, 3.56 and 5.61, respectively), indicating greater stability and adaptation (Table 3). On the other hand, those that showed the least adaptability with the highest Pim values were TBIO Sintonia and TBIO Sossego with 10.26 and 10.65, respectively.

**Figure 2:**
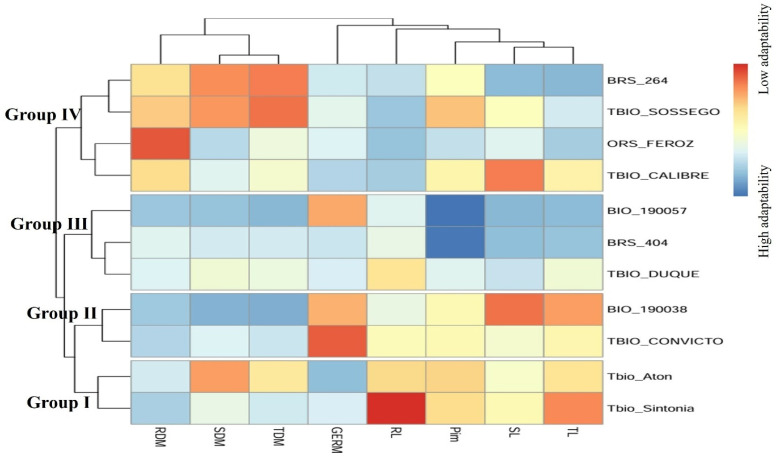
Heatmap for univariate (*Pi*) and multivariate (*Pim*) adaptability and stability values of the germination (GERM), shoot length (SL), root length (RL), total length (TL), shoot dry mass (SDM), root dry mass (RDM) and total dry mass (TDM) of 11 tropical wheat cultivars subjected to non-stressful (control) and stressful (drought and salinity) growth environments.

Another way to select the cultivars is through a cluster analysis using the means obtained in Table 3, and with them, a hierarchical grouping UPGMA (unweighted pair group method with arithmetic mean) was performed (Figure 2). The figure shows the formation of four different groups when considering the values obtained from the univariate *Pi* for the seven morphological traits evaluated and the multivariate *Pim*. The groups within Group III are those that show greater adaptability and stability, with the cultivars BIO 190057, BRS 404 and TBIO Duque being grouped together. This result confirms the selection in Table 2, which shows that the cultivars BIO 190057 and BRS 404 respond best to the applied drought and salinity stress.

**Figure 3:**
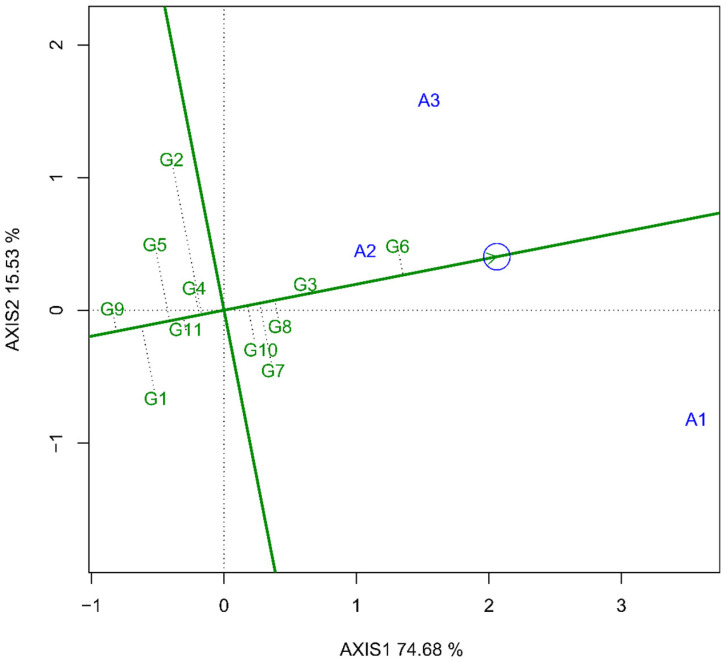
GGE biplot with information on the mean adaptability and stability for the data on germination, shoot length, root length, total length, shoot dry mass, root dry mass, and total dry mass of 11 wheat genotypes [TBIO Aton (G1), TBIO Sintonia (G2), BRS 404 (G3), BRS 264 (G4), TBIO Calibre (G5), BIO 190057 (G6), TBIO Duque (G7), BIO 190038 (G8), TBIO Sossego (G9), ORS Feroz (G10), TBIO Convicto (G11)], subjected to three abiotic stress environments [control (A1), salinity (A2) and drought (A3)].

In Figure 3, the results of the multivariate analysis of adaptability and stability via the GGE biplot are presented. As shown in Figure 3, the cultivars BIO 190057 and BRS 404 presented the greatest adaptability and stability simultaneously, which corroborates the results obtained from previous analyses and confirms the superiority of these two cultivars. In Figure 3 there is a significant distance between the points representing drought stress (A2) and salinity stress (A3). Some genotypes possess more robust defense mechanisms against drought stress, whereas others are more resistant to salinity stress. However, the genotypes BIO 190057 (G6) and BRS 404 (G3) exhibited defenses against both stresses while also demonstrating high performance under optimal germination conditions.

## 4. Discussion

The adverse environmental conditions faced by wheat crops in the world’s main producing regions require research to identify cultivars with greater tolerance and adaptation to these challenging environments. This study aimed to meet this need by evaluating tropical wheat cultivars under controlled drought and salinity conditions. Responses of wheat cultivars to aluminum toxicity, drought and salinity were investigated in the studies by Pereira et al. [23], Aguilera et al. [20], Aguilera et al. [24] and Argentel-Martínez et al. [25], which confirmed the existence of wide genetic variability in tolerance to the main abiotic stresses. This genetic variability for some of the main Brazilian tropical wheat cultivars was investigated in this study.

Figure 1 shows that the control environment resulted in the highest values for all evaluated traits, confirming that the two applied stresses can reduce the performance of the evaluated wheat cultivars. In general, during exposure to abiotic stresses, plants present a wide range of responses at the molecular, genetic and cellular levels, including physiological, morphological and developmental changes [6,7,26], as well as alterations in metabolic pathways [27] and gene expression [8,9], among other behaviors.

Seed germination is an important characteristic for most plant species, and adverse conditions of drought and salinity similarly limit the germination rate of wheat seeds. Ribeiro et al. [28] also reported that abiotic stresses reduced the germination of the 23 Brazilian wheat cultivars tested; however, these authors observed that wheat germination was higher than 85%, values higher than those obtained in the present study (75%). The optimal germination rate for wheat seeds should be greater than 85% [15], which highlights the negative impact of drought and salinity stresses, which reduce wheat germination by 15% (Figure 1). Although the weight of 1000 seeds varied between wheat cultivars (Table 1), it had no direct effect on the germination rate and initial growth of wheat plants (Table 3).

The same behavior was obtained for the variables SDM and TDM (Figure 1), showing that the magnitude of the reduction in the behavior of the variable was similar when drought and salinity stress were applied. Wheat crops can tolerate water stress without reducing seed germination; however, shoot (SDM and TDM) and root (RL and RDM) growth is drastically reduced by the highly negative osmotic potential promoted by water stress [29].

Drought stress was more severe when the behavior of wheat cultivars was evaluated for the variables SL and TL, revealing the detrimental effects that are physiologically obtained when plant growth is affected, especially in the aerial parts of the plants. The lack of water for plants results in water deficit, which, depending on the magnitude and timing, can be irreversible and can cause the death of the plant or the plant can be recovered if the magnitude and duration of the deficit are small. Wheat is a crop that tolerates water deficit, which often facilitates its cultivation in regions where water availability is limited. The selection of tolerant cultivars is always necessary to better respond to the abiotic stress present in most producing regions in Brazil [30,31,32], which provides an ecological and economic benefit [24]. Of the genotypes tested in this study, BIO 190057 stood out in the variables SL, TL, SDM, RDM and TDM, evidencing the high performance that this genotype has when subjected to water stress, in relation to the remaining genotypes.

Saline stress, in turn, was more severe when the variables RL and RDM were evaluated. Salinity stress is characterized by limited root development and, therefore, limited plant development. Brazil has stood out for generating genotypes with broad resistance to salinity and resistance to Al^3+^ [10,12,14,20,24,25]. The wide distribution of saline soils in the world makes the search for genotypes resistant or tolerant to these conditions a permanent demand [12]. Brazil stands out in this regard by providing genotypes such as BH-1146, internationally recognized as resistant to these stressful conditions, which values new genotypes that have maintained this characteristic through improvement [12,15,16,19,20]. Among the 11 genotypes tested, the BIO 190057 genotype also showed the best performance for saline stress, with superiority in five of the seven variables evaluated, which highlights its superior performance.

The greatest difficulty faced by breeders is selection in various environments or under conditions of abiotic or biotic stress. The wide response of genotypes to different climate, environmental, pest and disease conditions makes the selection process a difficult task. The response of genotypes is not always evident depending on the characteristic to be evaluated, which makes the selection process difficult. Among the methods proposed for selection that consider the best adaptability and stability capacity in favorable and unfavorable environments, the Lin and Binns [19] method was used. The method proposes an index from which values from zero to infinity are obtained, and the lower the values are, the greater the individual’s (genotype’s) capacity to have greater adaptability and stability. The univariate index (*Pi*) is obtained for each of the characteristics that can be evaluated in a given experiment, which, when considered independently, can make the selection process of superior individuals difficult. To eliminate this limitation of the method, Teodoro et al. [32] and Aguilera et al. [20] proposed the creation of an index multivariate Pi (*Pim*) that takes into account all variables and thus improves the process of selecting superior individuals.

The results of the application of the Lin and Binns [19] method revealed that, as expected, there was variation in the behavior of the 11 cultivars in relation to the seven characteristics evaluated (Table 2). When considering the germination, the three best genotypes were Tbio Aton, TBIO Calibre and BRS 404, with values of 0, 13.2 and 21.4, respectively. For the variables SL and TL, although infrequent, the same genotypes (BIO 190057, BRS 264 and BRS 404) remained for both variables (Table 3). For the RL variable, the selection process considering the univariate Pi values of Lins and Binns allows us to describe the genotypes ORS Feroz, TBIO Sossego and TBIO Calibre with values of 0.026, 0.0629 and 0.163, respectively, as those that had the least influence on the applied stresses promoted the greatest amount of root growth (Table 3).

When the univariate Pi values of the variables related to dry matter accumulation (SDM, RDM and TDM) were observed, the genotypes BIO 190038 and BIO 190057 were superior for these three variables, with the lowest index values and thus superiority in relation to the adaptability and stability proposed by Lins and Binns [19]. The results of the univariate *Pi* index value show how difficult it is to select because only in two groups of variables was the selection of the same genotype considered (Table 3). When we observe the multivariate index Pim, we can select those genotypes that stand out for more than one univariate index Pi, thus selecting the best genotypes with the capacity to better resist the stress conditions proposed in this work. The genotypes BIO 190057, BRS 404 and ORS FEROZ presented values of 3.45, 3.57 and 6.18, respectively. The genotype BIO 190057 is proposed as the best when Pim is considered; it is superior in the variables SL, TL, SDM, RDM and TDM, which represent 71% of the measured variables (Table 3).

Another way to select the genotypes is through a cluster analysis using the means obtained in Table 2, and with them, a hierarchical grouping UPGMA (unweighted pair group method with arithmetic mean) was performed (Figure 2). The figure shows the formation of four different groups when considering the values obtained from the univariate Pi for the seven characteristics evaluated and the multivariate Pim. The groups within Group III are those that show greater adaptability and stability, with the genotypes BIO 190057, BRS 404 and TBIO Duque being grouped together. This result confirms the selection in Table 3, which shows that the genotypes BIO 190057 and BRS 404 respond best to the applied water and salinity stress.

In Figure 3, the results of the multivariate analysis of adaptability and stability via the GGE biplot are presented. This analysis, according to Yan et al. [33] and Yan [28], allows for a clear visualization of these two characteristics. In the graph, the line with an arrow passing through the origin of the biplot indicates the direction where genotypes with greater adaptability are found, whereas the dashed lines perpendicular to this line indicate the stability of the genotypes in both directions. The longer the vector is associated with a particular genotype, the lower its stability [34].

As shown in Figure 3, the genotypes BIO 190057 and BRS 404 presented the greatest adaptability and stability, which corroborates the results obtained from previous analyses and confirms the superiority of these two cultivars. Another relevant point presented in Figure 3 is the significant distance between the points representing drought stress (A2) and salinity stress (A3). This difference indicates that although both stresses are related to water availability limitations for wheat seedlings, the defense mechanisms of the seedlings against each stress are different. Some genotypes possess more robust defense mechanisms against drought stress, whereas others are more resistant to salinity stress. However, the genotypes BIO 190057 and BRS 404 exhibited defenses against both stresses while also demonstrating high performance under optimal germination conditions.

Our findings contribute to the dissemination of useful methodologies for wheat breeding, especially those aimed at identifying superior genotypes adapted and stable for cultivation in areas with water restriction or relatively high levels of salinity and aluminum in the soil, at early stages of plant development. The resistance of crops to abiotic stress conditions is a current need to address the impacts of climate change and to allow cultivation in new areas where stress is limiting the crop. New experiments could be developed with the best genotypes selected here, which would allow verifying the power of early selection for the two abiotic stresses evaluated and correlating them with field responses. Accessing the genes responsible for the response is also of interest for future research [35,36]. Plant breeding, which is always concerned with the search for superior genotypes, uses the selection tools proposed in this work as excellent ways to select genotypes that are adaptable and stable to more than one abiotic stress, thus contributing to higher agricultural yields in unfavorable environments, which is essential for increasing global food security.

## 5. Conclusions

Eleven tropical wheat cultivars under drought and salinity stress conditions were investigated. Using univariate and multivariate approaches, we identified cultivars with high adaptability and stability based on the morphological traits of the wheat plants. The wheat cultivars BIO 190057, BRS 404 and TBIO Duque combine adaptability and stability for all morphological traits simultaneously. These genotypes can be grown both under non-stressful conditions and under drought and salinity stress conditions, as tested here. Furthermore, from a plant breeding perspective, these genotypes can be used as parents in wheat crossing blocks to obtain genotypes resistant to drought and salinity stresses.

## Figures and Tables

**Table 1:** Characterization of 11 tropical wheat cultivars for maturation cycle, water content, 1000-seed weight, seed germination rate and plant emergence rate.

Wheat Cultivar	Maturation Cycle	Water Content (%)	1000-Seed Weight (g)	Seed Germination (%)	Plant Emergence (%)
TBIO ATON	Medium	12.7	37.88 b	95 a	100 a
TBIO SINTONIA	Early	12.5	32.55 e	86 a	82 d
BRS 404	Early/Medium	12.8	38.73 b	89 a	92 b
BRS 264	Super Early	12.7	36.13 d	92 a	100 a
TBIO CALIBRE	Super Early	12.5	35.05 d	88 a	100 a
BIO 190057	Medium/Early	12.6	37.08 c	83 b	100 a
TBIO DUQUE	Early	12.9	34.68 d	91 a	100 a
BIO 190038	Early	12.7	37.20 c	79 b	100 a
TBIO SOSSEGO	Medium	12.6	34.90 d	88 a	100 a
ORS FEROZ	Super Early	12.5	40.10 a	94 a	100 a
TBIO CONVICTO	Medium/Late	12.8	40.33 a	82 b	84 c

Mean followed by distinct letters on the columns for the wheat cultivars show significant differences by the Scott–Knott test at the 0.05 level of confidence.

**Table 2:** Analysis of joint variance for the germination (G), shoot length (SL), root length (RL), total length (TL), shoot dry mass (SDM), root dry mass (RDM) and total dry mass (TDM) evaluated in 11 Brazilian wheat cultivars subjected to three abiotic stress environments.

Sources of Variation	DF	Probability > F						
		G	SL	RL	TL	SDM	RDM	TDM
Genotypes (G)	10	0.000	0.000	0.000	0.000	0.000	0.000	0.000
Environments (E)	2	0.000	0.000	0.000	0.000	0.000	0.000	0.000
G × E	20	0.282	0.212	0.001	0.051	0.025	0.481	0.076
Mean		79.23	2.07	5.34	17.41	12.11	3.98	16.08
CV (%)		7.64	9.95	11.29	7.85	9.21	9.92	8.36

Abbreviations: CV, coefficient of variation; DF, degrees of freedom.

**Table 3:** Univariate (*Pi*) and multivariate adaptability and stability (*Pim*) values for the germination (G), shoot length (SL), root length (RL), total length (TL), shoot dry mass (SDM), root dry mass (RDM) and total dry mass (TDM) of 11 tropical wheat cultivars subjected to non-stressful (control) and stressful (drought and salinity) growth environments.

Cultivar	*Pi* Values—Lin and Binns (1988)																					
	G			SL			RL			TL			SDM			RDM			TDM			*Pim*
	D *	S	*Pi*	D	S	*Pi*	D	S	*Pi*	D	S	*Pi*	D	S	*Pi*	D	S	*Pi*	D	S	*Pi*	
BIO 190038	99	109	4.70	6	5	5.40	1	0.5	1.47	8	7	4.57	0	0	0.00	0.01	0.01	0.05	0	0	0.00	8.31
BIO 190057	58	128	4.11	0.03	0.03	0.03	0.6	0.5	1.17	0.03	0.1	0.04	0.4	0.4	0.35	0.002	0.001	0.01	0.4	0.4	0.21	3.08
BRS 264	22	14	0.83	0.003	0.07	0.04	0.5	0.3	0.81	0.03	0.03	0.02	7	6	5.68	0.8	0.7	3.89	12	11	6.04	8.72
BRS 404	20	20	0.91	0.001	0.2	0.10	1	0.5	1.47	0.2	0.3	0.15	2	2	1.75	0.3	0.1	1.03	3	3	1.58	3.56
ORS Feroz	36	6	0.99	2	1	1.48	0	0.04	0.06	0.5	0.8	0.40	1	1	0.88	1	1	5.19	4	4	2.10	5.61
TBIO Aton	0	0	0.00	3	3	2.94	3	1	3.72	7	6	3.96	6	5	4.80	0.2	0.1	0.77	7	5	3.15	9.80
TBIO Calibre	17	14	0.71	5	6	5.39	0.2	0.1	0.29	5	6	3.37	2	2	1.75	0.8	0.7	3.89	5	5	2.63	9.12
TBIO Convicto	132	116	5.64	3	2	2.46	2	1	2.95	6	3	2.71	2	2	1.75	0.1	0.08	0.47	3	2	1.31	8.88
TBIO Duque	3	39	0.90	1	1	0.98	2	2	4.35	4	3	2.13	2	1	1.29	0.2	0.2	1.04	3	2	1.31	6.12
TBIO Sintonia	24	34	1.30	3	4	3.43	4	2	5.89	9	9	5.50	2	3	2.22	0.05	0.03	0.21	3	3	1.58	10.26
TBIO Sossego	17	44	1.34	3	3	2.94	0.09	0.04	0.13	2	2	1.22	6	6	5.26	0.9	0.8	4.41	11	11	5.79	10.65

* Average of the notes and estimates of *Pi* are shown for each of the abiotic stress environments (D: drought and S: salinity).

## Data Availability

The data are in the body of the work.
